# miR-29c-5p knockdown reduces inflammation and blood–brain barrier disruption by upregulating LRP6

**DOI:** 10.1515/med-2022-0438

**Published:** 2022-02-22

**Authors:** Qijun Dai, Jian Sun, Tianyi Dai, Qin Xu, Yueqin Ding

**Affiliations:** Department of Neurology, Haian Hospital of Traditional Chinese Medicine, Haian, 226600, China; Department of Endocrinology, Jingjiang Hospital of Traditional Chinese Medicine, Jingjiang, 214500, China; College of Traditional Chinese Medicine, Nanjing University of Chinese Medicine, Class 1802, Nanjing, 210023, China; Department of Nursing, Haian Hospital of Traditional Chinese Medicine, Haian, 226600, China

**Keywords:** ischemic stroke, blood–brain barrier, brain microvascular endothelial cells, astrocytes, miRNA

## Abstract

Blood–brain barrier participates in the pathological process of ischemic stroke. MicroRNA-29c-5p was highly expressed in clinical samples from patients with ischemic stroke. In this study, oxygen-glucose deprivation (OGD) treatment of astrocytes enhanced the permeability of brain microvascular endothelial cells (BMECs), and the miR-29c-5p expression was elevated in clinical samples from patients with ischemic stroke. For the function of miR-29c-5p in ischemic stroke, the miR-29c-5p knockdown decreased the permeability and the tight junction protein (TJP) destruction of BMECs and ameliorated the inflammation induced by OGD-treated astrocytes. Mechanistically, miR-29c-5p interacted with lipoprotein receptor-related protein 6 (LRP6) and negatively regulated the LRP6 expression in astrocytes. Moreover, the rescue assays indicated that the interference with miR-29c-5p ameliorated the TJP destruction of BMECs and inflammation caused by OGD-treated astrocytes by increasing the LRP6 expression. Together, miR-29c-5p knockdown decreased the high permeability and the TJP destruction of BMECs and ameliorated the inflammation induced by OGD-treated astrocytes by elevating LRP6 expression.

## 1 Introduction

Ischemic stroke is a central nervous system disease with high morbidity and mortality worldwide [1]. One of the pathophysiological features of ischemic stroke is the destruction of blood–brain barrier (BBB) [2]. BBB is formed by the tight junctions and adhesion of endothelial cells, and it acts as a functional barrier between the blood and the brain, which provides an optimal environment for neural network [3]. Considering the critical function of BBB dysfunction in the pathophysiology of ischemic stroke [4,5], maintaining BBB function and integrity are expected to alleviate brain injury after ischemic stroke. However, the mechanisms that regulate the BBB function after ischemic stroke have not been fully established.

Brain microvascular endothelial cells (BMECs) and astrocytes are major components of BBB [6]. In brain injury, BMECs and astrocytes support the formation of a neurovascular unit, a complex structural and functional unit that protects the BBB [7,8]. As the most abundant cell type in the central nervous system, astrocytes exert pivotal functions in maintaining normal brain function [9]. BMECs exert a critical role in BBB function through a highly selective barrier, in which tight junction proteins (TJPs) such as claudin-5, occludin, and zonula occluden-1 (ZO-1) are vital proteins regulating BBB integrity [10,11]. Under physiological and pathological conditions, BMECs–astrocyte interactions and signal transduction are critical for BBB integrity [12]. Thus, uncovering the underlying mechanism of BMECs–astrocyte interaction is essential for protecting BBB function after ischemic stroke.

MicroRNAs (miRNAs) are small noncoding RNAs that regulate mRNA degradation or translation by binding to the 3′-untranslated region (UTR) of target mRNAs [13]. MiRNAs participate in regulating various cellular behaviors, mainly including cell proliferation, differentiation, and metabolism [14,15]. Recently, increasing miRNAs have been confirmed to be abnormally expressed in ischemic stroke and to mediate the occurrence and development of ischemic stroke. For instance, Zuo et al. found that miR-132 alleviates brain injury by protecting the BBB in mice, suggesting that miR-132 might be a novel therapeutic target for BBB protection in ischemic stroke [16]; Zhang et al. indicated that miR-182 protects the integrity of the BBB during cerebral ischemia by repressing endothelial apoptosis, implying that miR-182 might be a potential target for the treatment of BBB disruption during cerebral ischemia [17]. Notably, another study confirmed that in an *in vitro* BBB model induced by the coculture of BMECs and astrocytes, miR-9-5p alleviates BBB injury and neuroinflammatory response and promotes neurological function recovery [18]. Thus, the discovery of novel miRNAs involved in the BMECs–astrocyte interaction is beneficial to better reveal the regulatory mechanism of BBB function after ischemic stroke.

The low-density lipoprotein receptor-related protein (LRP) signaling pathway participates in several processes, such as cerebral vascular remodeling and cerebral ischemia [19]. LRP6 is a single-span transmembrane protein and is involved in the regulation of various disease processes, including ischemic stroke [20]. As has been reported, the mutations in LRP6 are associated with the risk of ischemic stroke [21]; LRP6 protects the brain from ischemic injury, suggesting that LRP6 might provide a novel therapeutic approach for ischemic stroke [22]. In this study, the abnormal expressions of the miR-181 and miR-29 families in cerebral ischemia astrocytes were verified in the clinical samples from patients with ischemic stroke. Furthermore, the results authenticated that only miR-29c-5p was markedly overexpressed. Based on this finding, we selected miR-29c-5p as the mainly studied miRNA. Meanwhile, we found the binding sites between miR-29c-5p and LRP6 through TargetScan and Metascape databases. On this basis, we used various molecular techniques to conduct in-depth studies on its role and mechanism in the BBB after ischemic stroke, aiming to provide novel potential biomarkers for the clinical treatment of ischemic stroke.

## 2 Materials and methods

### 2.1 Clinical samples

Twenty patients with ischemic stroke and the matched healthy controls were included in this research. All of them were diagnosed based on a detailed stroke history and a physical examination using a professional clinical care provider. After obtaining informed consent from the patient or family members, the blood samples from the patients were collected for subsequent research. This study was approved by the Ethics Committee of Haian Hospital of Traditional Chinese Medicine.

### 2.2 Quantitative real-time PCR (qRT-PCR)

The expressions of miR-29c-5p, miR-29a-5p, miR-181-5p, and LRP6 in the blood samples of patients with ischemic stroke, and the LRP6 expression in astrocytes were determined using qRT-PCR. In brief, Trizol reagent (ThermoFisher Scientific, WA, USA) was used to extract the total RNAs, and the concentrations of RNAs were quantified by a NanoDrop spectrophotometer (Nanodrop Technologies, USA). To measure the LRP6 expression, High-Capacity cDNA Reverse Transcription Kit (Thermo Fisher Scientific) was used. To assess the expressions of miR-29c-5p, miR-29a-5p, and miR-181-5p, TaqMan MicroRNA Reverse Transcription Kit (Thermo Fisher Scientific) was used. Subsequently, the real-time PCR was conducted on a 7500 Real-Time PCR system (Applied Biosystems, USA) with TaqMan Universal PCR Master Mix (Thermo Fisher Scientific). The expressions of miR-29c-5p, miR-29a-5p, and miR-181-5p were normalized to U6, and the *LRP6* expression was normalized to GAPDH. The relative expression of the above molecules was determined by the 2^−ΔΔCT^ method. All the primer sequences are exhibited in Table 1.

**Table 1:** The sequences of all primers used in qRT-PCR

Gene name	Primer sequence (5′–3′)
miR-29c-5p	Forward: TAGTAGTGGTTGTTTGTTTTTTTGA
	Reverse: CCACTCTACTAAAAACTCCATCTCC
miR-29a-5p	Forward: GCGGCGGACTGATTTCTTTTGGT
	Reverse: ATCCAGTGCAGGGTCCGAGG
miR-181-5p	Forward: ACACTCCAGCTGGGAACATTCAACGCTGTCGG
	Reverse: TGGTGTCGTGGAGTCGA
LRP6	Forward: TCAACCCAGAGCTATTGCCTT
	Reverse: TAACCACTGCCTGCCGATTT
U6	Forward: GCTTCGGCAGCACATATACTAAAAT
	Reverse: CGCTTCACGAATTTGCGTGTCAT
GAPDH	Forward: CTCTGACTTCAACAGCGAC
	Reverse: CGTTGTCATACCAGGAAATGAG

### 2.3 Isolation and identification of primary astrocytes

Primary astrocytes were obtained from neonatal 1-day-old C57BL/6 mice [23,24]. In detail, the cortex was isolated from the mouse brain, and the meninges were removed. Subsequently, the cortex was digested at 37°C with 0.25 mg/mL trypsin and 0.1 mg/mL DNase for 20 min. The resuspended cells were collected and placed in a growth medium supplemented with high-glucose Dulbecco’s Modified Eagle’s Medium (DMEM, Thermo Fisher Scientific) with 10% fetal bovine serum (FBS, Thermo Fisher Scientific) and 1% penicillin–streptomycin, and then placed in a polylysine-coated flask and grown at 37°C, in the presence of 5% CO_2_. The plastic flask was then placed on a shaker and gently shaken at 260 rpm to separate the microglia. The remaining adherent astrocytes were identified using immunofluorescence staining, and glial fibrillary acidic protein (GFAP) was used as a molecular marker, and more than 95% of the cells were positive for GFAP.

### 2.4 Cell culture and different treatments

Primary BMECs (5 × 10^5^ cells) were purchased from Procell (Wuhan, China). The cells were put in 90% high-glucose DMEM containing 10% FBS and cultured at 37°C, in the presence of 5% CO_2_.

After washing the astrocytes twice with PBS, the cells were placed in oxygen-glucose deprivation (OGD) medium (glucose-free and serum-free DMEM with 10 μM hemin) and cultured in hypoxia (5% CO_2_ + 0.4% O_2_ + 95% N_2_) at 37°C for 4 h.

To initiate the coculture system of BMECs and astrocytes, primary astrocytes isolated from normal mice were treated with OGD for 3 h. Subsequently, BMECs were transferred to a medium containing primary astrocytes for coculture for 24 h [25].

### 2.5 Transepithelial electrical resistance (TEER) analysis

A Millicell-ERS instrument (Millipore, MA, USA) was used to detect the TEER values of BMECs. In detail, the isolated primary astrocytes (1 × 10^4^ cells) were grown on six-well plates for 2 days. Then, the coculture inserts were placed at room temperature for 25 min. Subsequently, we detected the values of TEER immediately after changing the medium. Before calculating the final resistance, the background resistance was subtracted and the TEER value was expressed as Ω cm^2^.

### 2.6 Analysis of the penetration rate of sodium fluorescein (NaF)

To assess the barrier formation, we measured the exclusion of NaF daily for 4 days. In brief, we added 5 µM NaF (Sigma-Aldrich, Buchs, Switzerland) to the apical chamber and then added 1 mL standard medium to the basal chamber. After incubating for 2 h, we measured the NaF in basolateral samples by a microplate reader (Berthold Technologies GmbH, excitation 485 nm, emission 528 nm).

### 2.7 Cell transfection

miR-29a-5p inhibitor, negative control (NC)-inhibitor, miR-29a-5p mimic, NC-mimic, si-LRP6, and si-NC were synthesized from GenePharma (Shanghai, China).

The cell transfection was conducted using Lipofectamine 2000 (Thermo Fisher Scientific). In particular, the astrocytes (3 × 10^5^ cells) were grown in six-well plates for 24 h. Subsequently, the miR-29a-5p inhibitor, miR-29a-5p mimic, si-LRP6, and their corresponding controls were transfected into astrocytes using Lipofectamine 2000. Forty-eight hours after transfection, we collected the astrocytes for subsequent studies.

### 2.8 Western blot assay

BMECs and primary astrocytes with different treatments were harvested and radioimmunoprecipitation assay (RIPA) lysis buffer (Thermo Fisher Scientific) was used to isolate the total proteins. The same amount of protein samples were separated using 10% sodium dodecyl sulfonate-polyacrylamide gel electrophoresis and transferred onto membranes (Millipore, Massachusetts, USA). Then, the membranes were incubated with 5% skim milk for 1 h. The membranes were further incubated with anti-GFAP (Abcam, 1:10,000 dilution, ab7260), anti-GLP-1R (Abcam, 1:1,000 dilution, ab218532), anti-claudin-5 (Abcam, 1:1,000 dilution, ab131259), anti-occludin (Abcam, 1:1,000 dilution, ab216327), anti-ZO-1 (Abcam, 1:1,000 dilution, ab276131), anti-VEGF-A (Abcam, 1:1,000 dilution, ab214424), anti-MMP-9 (Abcam, 1:1,000 dilution, ab76003) and anti-β-actin (Abcam, 1 µg/mL, ab8226) at 4°C overnight. Then, the membranes were further incubated with the secondary antibodies (Abcam, 1:2,000 dilution, ab205718). Ultimately, the signal of protein level was visualized by an enhanced chemiluminescent reagent (Thermo Fisher Scientific), and the images were analyzed by using Image-Pro Plus 5.1 Image Analysis Software (Media Cybernetics, MD, USA).

### 2.9 Lactate dehydrogenase (LDH) release assay

Referring to the standard procedure from reagent manufacturers, LDH release assay was performed using an LDH Cytotoxicity Assay Kit (Abcam, Cambridge, UK). BMECs with different treatments (3 × 10^3^ cells) were put in 96-well plates and cultured for 24 h. The cells were lysed using 0.2% Triton X-100 (Solarbio, Beijing, China). After centrifugation, the supernatants were collected and coincubated with LDH reaction solution. Subsequently, a microplate reader (Bio-Rad Co., Hercules, CA, USA) was conducted to detect the optical density (OD) value at 490 nm. The content of LDH in cell lysates was determined to assess the cellular injury.

### 2.10 Immunofluorescence staining assay

Based on the previously reported methods [26], the immunofluorescence staining was conducted to assess the expressions of TJPs claudin5, occludin, and ZO-1. The primary antibodies were anti-claudin5 (Abcam, Assay dependent, ab15106), anti-occludin (Abcam, 1:100 dilution, ab216327), anti-ZO-1 (Abcam, 1:100 dilution, ab221547), anti-VEGFA (Abcam, 1 µg/mL, ab39250), and anti-MMP-9 (Abcam, 1:500 dilution, ab76003). The secondary antibodies were Alexa Flur 488 mouse anti-rabbit IgG and tetraethyl rhodamine isothiocyanate goat anti-rabbit IgG. The nuclei were stained using 4′,6-Diamidino-2-phenylindole staining solution (Sigma-Aldrich). Ultimately, Vectra® Polaris™ Automated Quantitative Pathology Imaging System (PerkinElmer, USA) was used to analyze all images.

### 2.11 Enzyme-linked immunosorbent assay (ELISA)

The contents of inflammatory cytokines interleukin-1β (IL-1β), interleukin-6 (IL-6), Tumor Necrosis Factor Alpha (TNF-α), and Transforming growth factor β (TGF-β) in the cell culture supernatant of astrocytes were quantified by using ELISA. Briefly, astrocytes (3 × 10^3^ cells) were put in 96-well plates. The ELISA kit (ThermoFisher Scientific) was conducted to detect the concentrations of IL-1β, IL-6, TNF-α, and TGF-β in the cell culture supernatant of astrocytes based on the instructions of the manufacturer. The OD at 450 nm was measured.

### 2.12 Dual-luciferase reporter gene assay

Before the cell transfection, the astrocytes were grown in six-well plates and cultured to 80% fusion. The pmirGLO reporter vector containing a wild type (WT) or mutant type (Mut) miR-29a-5p binding sites in the 3′-UTR of LRP6, and named LRP6 WT or LRP6 Mut, was synthesized by Ribobio (Guangdong, China). miR-29a-5p mimic and the above recombinant vector were cotransfected into astrocytes using Lipofectamine 2000. Forty-eight hours after the transfection, the dual-luciferase reporter assay system (Thermo Fisher Scientific) was used to measure the relative luciferase activity.

### 2.13 Statistical analysis

All data were presented as the mean ± standard deviation. When the differences between the control and experimental groups were assessed, an unpaired Student’s *t*-test was carried out; when the differences among more than two groups were assessed, one-way analysis of variance (ANOVA) followed by Tukey’s post-test was conducted. *P <* 0.05 was considered significant.


**Ethics approval and consent to participate:** The experimental protocol was established, according to the ethical guidelines of the Helsinki Declaration and was approved by the Ethics Committee of Haian Hospital of Traditional Chinese Medicine.

## 3 Results

### 3.1 OGD treatment of astrocytes increases the permeability of BMECs

**Figure 1:**
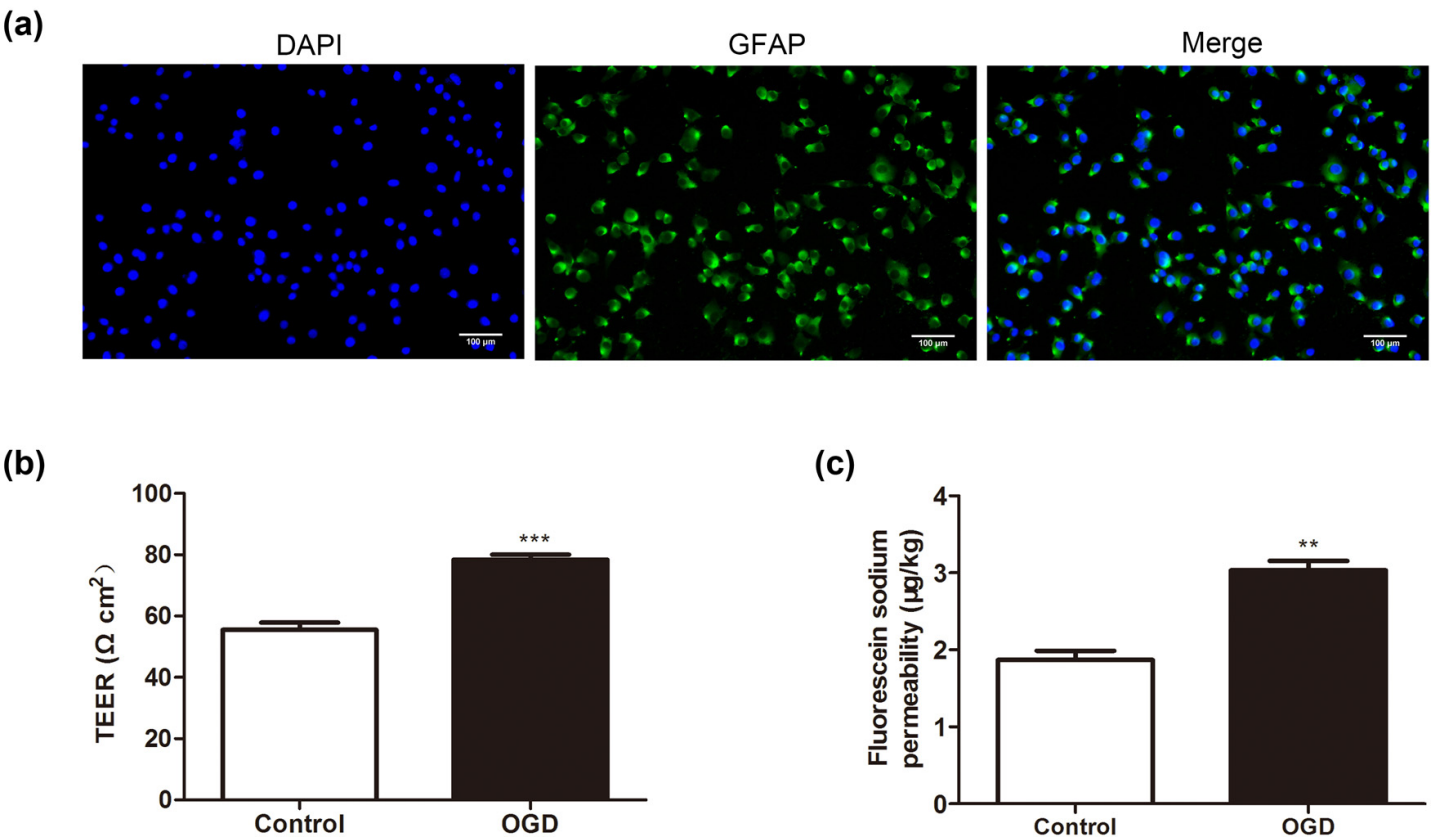
Influence of the OGD treatment of astrocytes on the permeability of BMECs. The primary astrocytes were isolated, and the primary astrocytes were treated with OGD for 3 h and then cocultured with primary brain microvascular epithelial cells (BMECs) for 24 h. (a) An immunofluorescence staining assay was performed to verify the isolated primary astrocytes (scale bar: 100 µm). (b) The TEER value of BMECs was determined using the Millicell-ERS instrument (Millipore, MA, USA). (c) Analysis of the penetration rate of NaF. OGD: oxygen-glucose deprivation, TEER: transepithelial electrical resistance, and NaF: sodium fluorescein. Data are presented as an average of three independent assays. **P < 0.01, ***P < 0.001 vs control.

BBB is essential for maintaining normal brain activity and is composed of BMECs arranged in blood vessels, and BMECs form a barrier to regulate the transportation of cells between the blood and the brain [27]. Moreover, the coculture of BMECs and astrocytes is commonly used as the *in vitro* model of BBB [18]. In this study, we isolated primary astrocytes from mice and identified them by immunofluorescence staining assay. GFAP was used as a molecular marker, and more than 95% of the cells were positive for GFAP (Figure 1a). Subsequently, the primary astrocytes treated with OGD were cocultured with BMECs. TEER analysis revealed that compared to the control group, OGD treatment increased the TEER of BMECs (Figure 1b). Similarly, compared to the control group, OGD treatment enhanced the ability of NaF to penetrate BMECs (Figure 1c). In general, OGD treatment of astrocytes enhanced the permeability of BMECs.

### 3.2 Low expression of miR-29c-5p reduces the high permeability of BMECs caused by OGD-treated astrocytes

We further detected the expressions of miR-29c-5p, miR-29a-5p, and miR-181-5p in the blood samples of patients with ischemic stroke, and the results indicated that only the miR-29c-5p was elevated, suggesting that miR-29c-5p might be related to the occurrence of ischemic stroke (Figure S1). Thus, miR-29c-5p was selected as the main miRNA for our subsequent studies.

**Figure 2:**
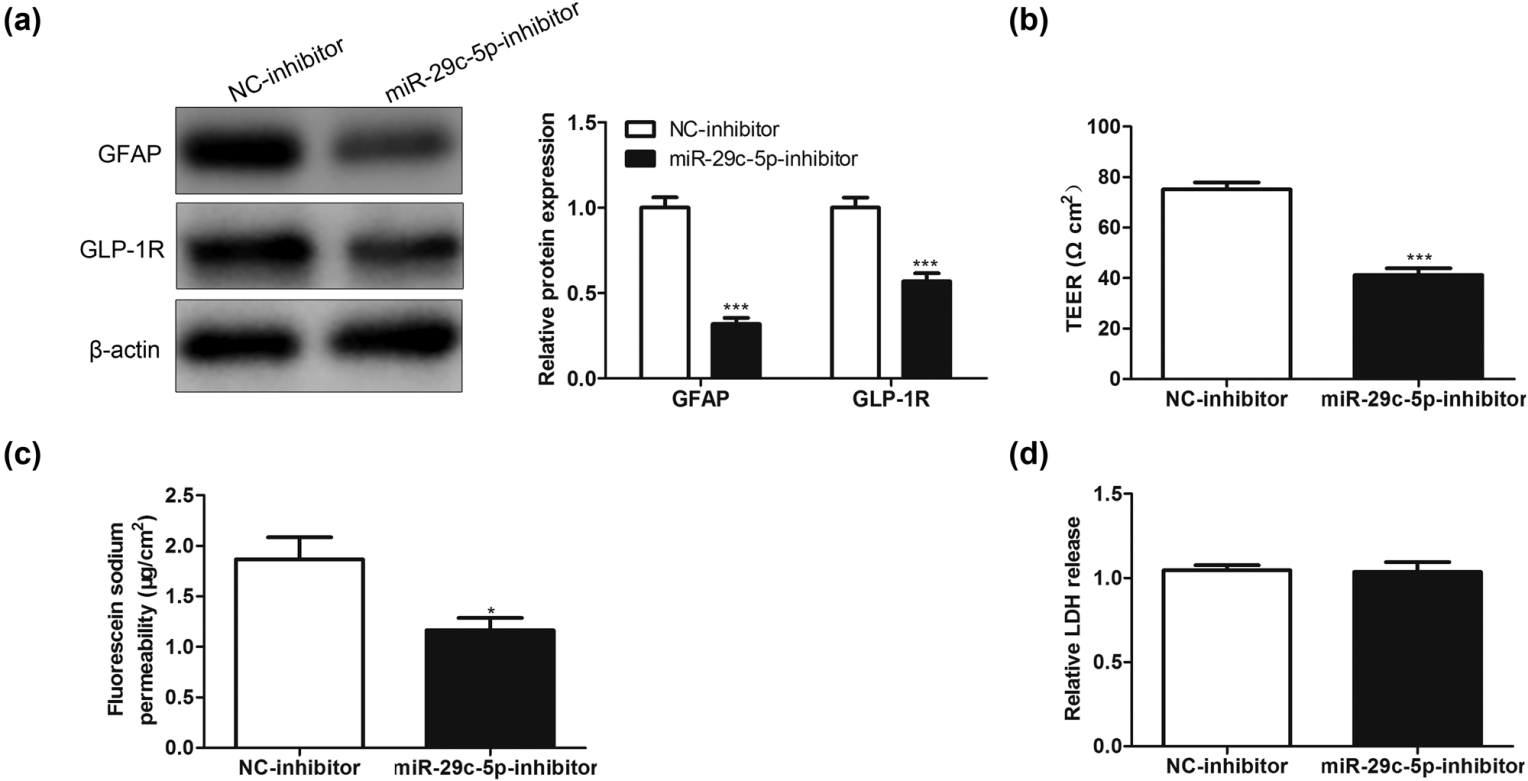
Effect of miR-29c-5p on the high permeability of BMECs caused by OGD-treated astrocytes. Astrocytes transfected with miR-29c-5p inhibitor or NC-inhibitor were treated with OGD for 3 h and then cocultured with BMECs for 24 h. (a) Western blot was performed to analyze the protein levels of astrocyte activation markers GFAP and GLP-1R. (b) Analysis of the TEER value of BMECs. (c) Detection of the ability of NaF to penetrate BMECs. (d) LDH release assay was performed to assess the injury of BMECs. NC: negative control, GLP-1R: glucagon-like peptide-1 receptor, and LDH: lactate dehydrogenase. Data are presented as an average of three independent assays. *P < 0.05, ***P < 0.001 vs NC-inhibitor.

Furthermore, we sought to explore whether miR-29c-5p mediated the high permeability of BMECs caused by OGD-treated astrocytes. Astrocytes transfected with miR-29c-5p inhibitor or NC-inhibitor were treated with OGD and then cocultured with BMECs. We detected the protein levels of astrocyte activation markers GFAP and glucagon-like peptide-1 receptor (GLP-1R) and found that compared to the NC-inhibitor group, the GFAP and GLP-1R protein levels were decreased in the miR-29c-5p inhibitor group (Figure 2a). Meanwhile, the TEER value in the miR-29c-5p inhibitor group was much lower than that in the NC-inhibitor group (Figure 2b). Similar to this finding, the ability of NaF to penetrate BMECs was weakened in the miR-29c-5p inhibitor group compared to the NC-inhibitor group (Figure 2c). Cell injury can be assessed through the LDH release assay [28]. As shown in Figure 2d, compared with the NC-inhibitor group, there was no remarkable change in the LDH release of BMECs in the miR-29c-5p inhibitor group. Together, the above results clarified that the miR-29c-5p knockdown decreased the high permeability of BMECs induced by OGD-treated astrocytes.

### 3.3 miR-29c-5p knockdown ameliorates TJP destruction of BMECs induced by OGD-treated astrocytes

**Figure 3:**
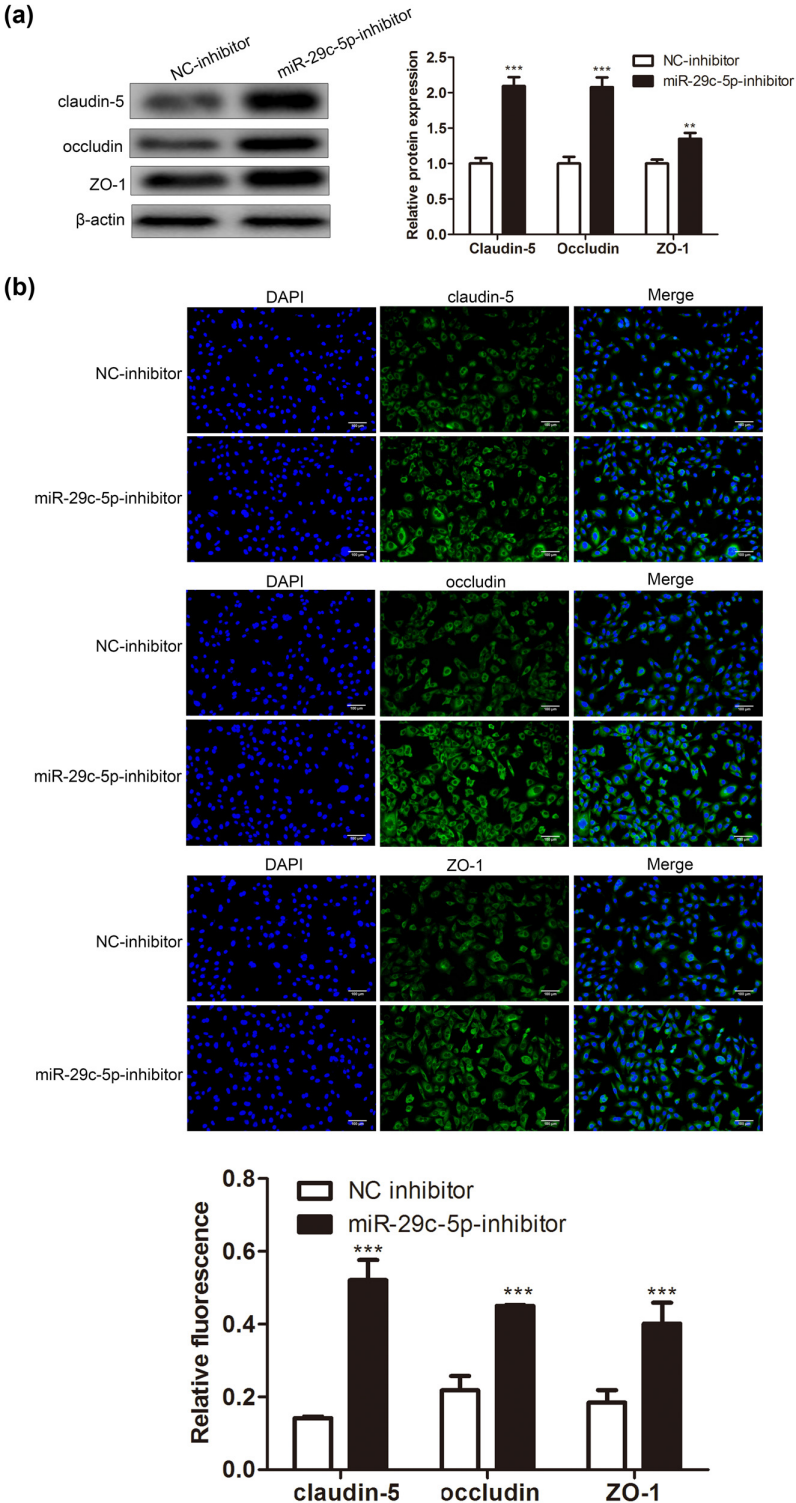
Influence of miR-29c-5p on the TJP destruction of BMECs caused by OGD-treated astrocytes. Astrocytes transfected with miR-29c-5p inhibitor or NC-inhibitor were treated with OGD for 3 h, and the cells were cocultured with BMECs for 24 h. (a) The protein levels of TJPs claudin-5, occludin, and ZO-1 were determined using Western blot. (b) Immunofluorescence staining assay was applied to quantify the claudin-5, occludin, and ZO-1 expressions (scale bar: 100 µm). Data are presented as an average of three independent assays. **P < 0.01, ***P < 0.001 vs NC-inhibitor.

Earlier studies indicate that one of the biochemical features of BBB injury is the decreased expression of TJPs, mainly including claudin-5, occludin, and ZO-1 [2,29]. Astrocytes transfected with miR-29c-5p inhibitor or NC-inhibitor were treated with OGD and then cocultured with BMECs. Western blot analysis authenticated that claudin-5, occludin, and ZO-1 were upregulated after the miR-29c-5p knockdown (Figure 3a). Immunofluorescence staining assay further confirmed that claudin-5, occludin, and ZO-1 expressions were increased in the miR-29c-5p inhibitor group (Figure 3b). In general, the interference with miR-29c-5p relieved the TJP destruction of BMECs caused by OGD-treated astrocytes.

### 3.4 Interference with miR-29c-5p relieves inflammation induced by OGD-treated astrocytes

**Figure 4:**
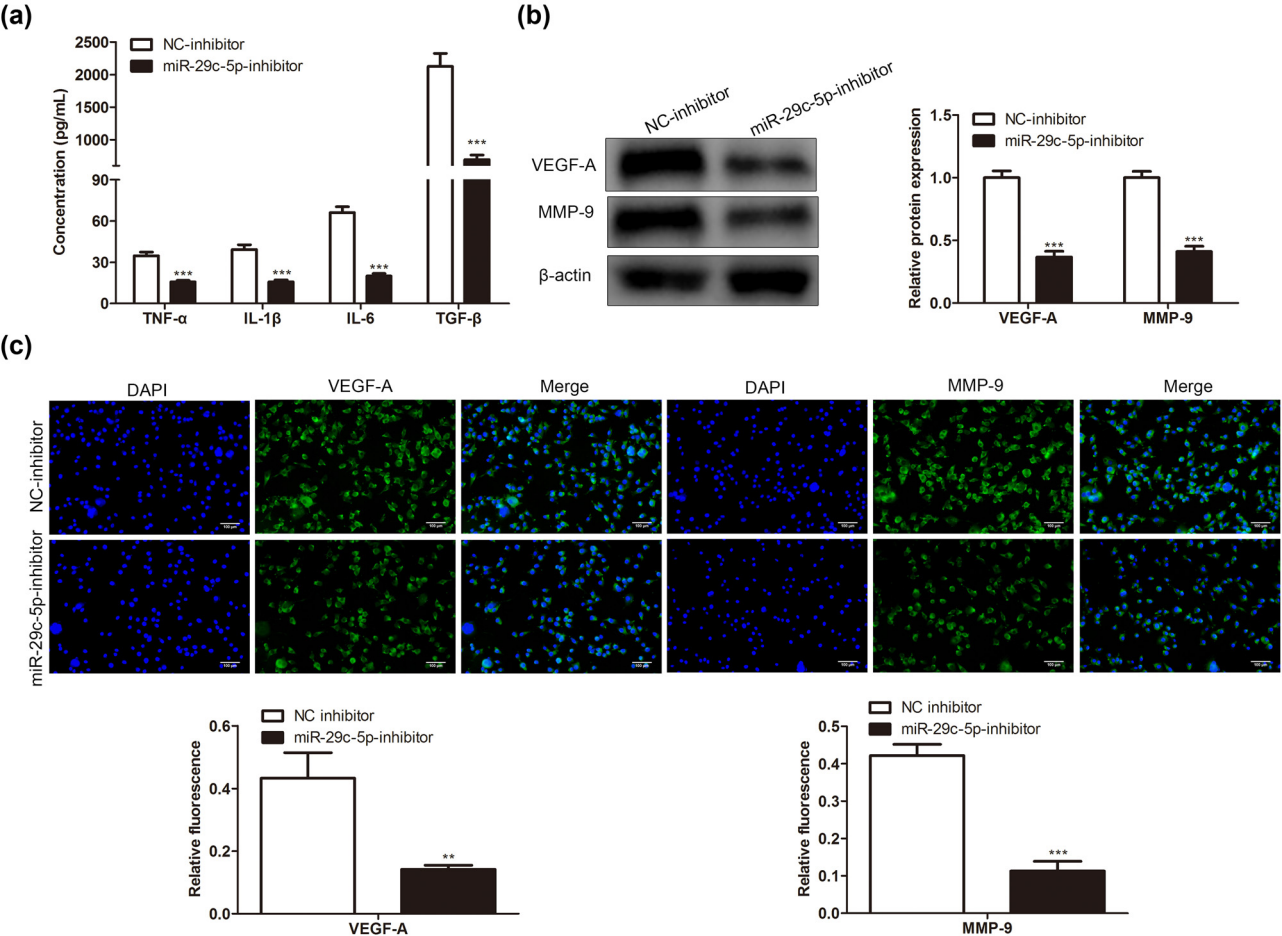
Effect of miR-29c-5p on the inflammation caused by OGD-treated astrocytes. Astrocytes transfected with miR-29c-5p inhibitor or NC-inhibitor were treated with OGD for 3 h. (a) ELISA was performed to test the concentrations of inflammatory cytokines IL-1β, IL-6, TNF-α, and TGF-β in cell culture supernatant of astrocytes. (b) The protein levels of inflammatory factors VEGF-A and MMP-9 were tested using Western blot. (c) An immunofluorescence assay was performed to assess the expressions of VEGF-A and MMP-9 (scale bar: 100 µm). Data are presented as an average of three independent assays. **P < 0.01, ***P < 0.001 vs NC-inhibitor.

Based on the above findings, we attempted to clarify whether miR-29c-5p mediated the inflammation induced by OGD-treated astrocytes. As exhibited in Figure 4a, the miR-29c-5p knockdown reduced the concentrations of IL-1β, IL-6, TNF-α, and TGF-β. Inflammatory factors such as vascular endothelial growth factor (VEGF-A) and matrix metalloproteinase-9 (MMP-9) directly or indirectly aggravate BBB [30,31]. As shown in Figure 4b, compared with the NC-inhibitor group, the protein levels of VEGF-A and MMP-9 were decreased in the miR-29c-5p inhibitor group. Furthermore, the immunofluorescence results showed the same trend (Figure 4c). Together, the miR-29c-5p knockdown reduced the inflammation caused by OGD-treated astrocytes.

### 3.5 MiR-29c-5p targets LRP6

**Figure 5:**
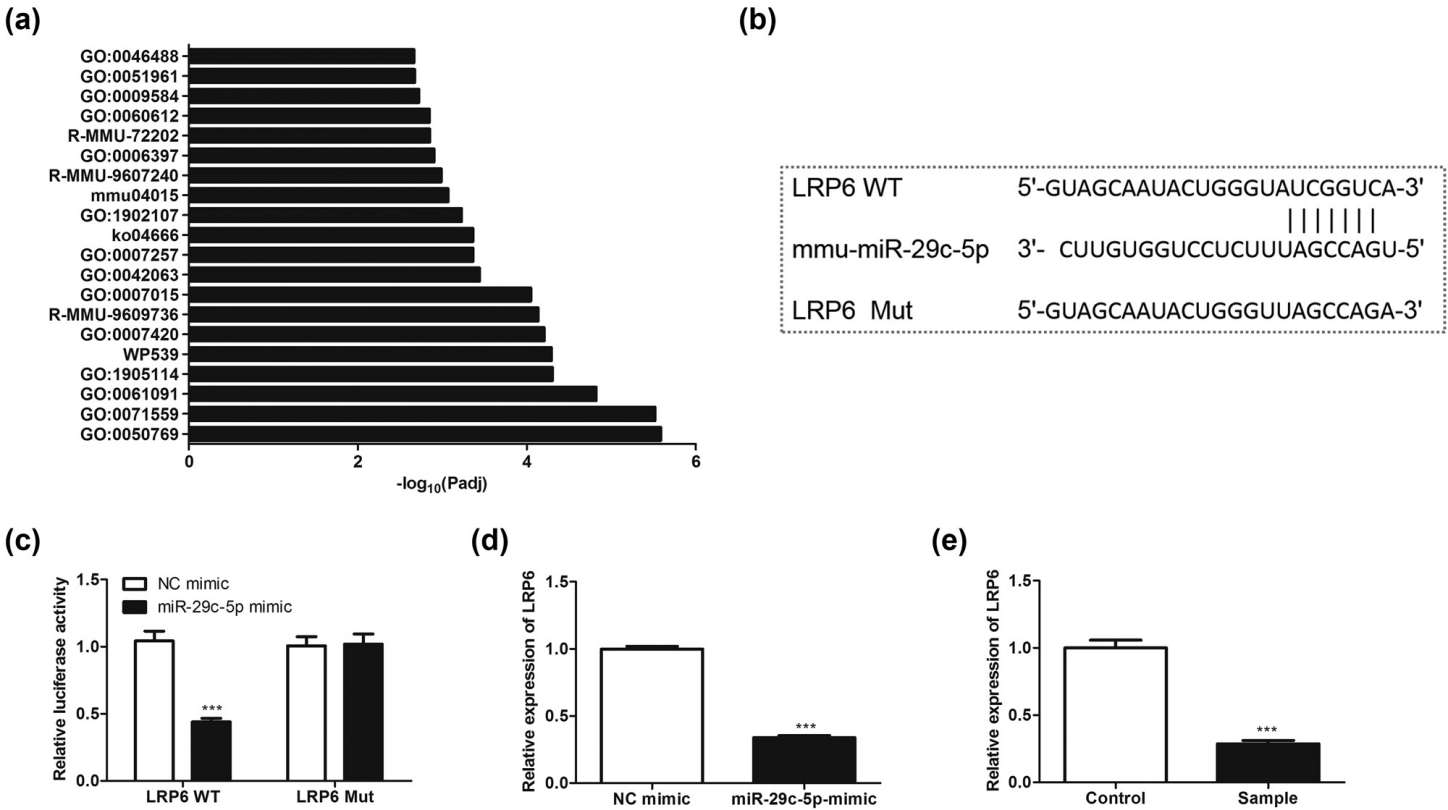
Verification of the interaction between miR-29c-5p and LRP6: (a and b) Bioinformatic databases, TargetScan and Metascape, were applied to predict the possible downstream regulatory targets of miR-29c-5p and screen out the targets that participated in regulating the Wnt pathway. (c) The binding of miR-29c-5p to LRP6 was proved by dual-luciferase reporter gene assay. (d) miR-29c-5p mimic or NC-mimic was transfected into astrocytes. QRT-PCR was carried out to quantify the LRP6 expression. (e) A total of 20 patients with ischemic stroke and 20 matched healthy controls were included, and the blood samples were isolated from them. The expression of LRP6 was analyzed by qRT-PCR. Data are presented as an average of three independent assays. ***P < 0.001 vs NC mimic.

Recent studies have confirmed that the activation of the Wnt/β-catenin signaling by protective astrocytes repairs BBB injury [32], and miRNAs exert regulatory functions by targeting the 3′-UTR of mRNAs [33]. Thus, we applied bioinformatic databases (TargetScan and Metascape) to predict the possible downstream regulatory targets of miR-29c-5p and screened out the targets that participated in the regulation of the Wnt pathway. The results showed that there were binding sites between miR-29c-5p and LRP6 (Figure 5a and b). To verify this combination, we performed the dual-luciferase reporter gene assay, and the results confirmed that the transfection of miR-29c-5p mimic reduced the relative luciferase activity of LRP6 WT, and had no obvious changes on the relative luciferase activity of LRP6 MUT, implying that there was an interaction between miR-29c-5p and LRP6 (Figure 5c). Subsequently, we transfected miR-29c-5p mimic into astrocytes to investigate the regulation of miR-29c-5p on the LRP6 expression. The results authenticated that the transfection of miR-29c-5p mimic decreased the LRP6 expression (Figure 5d). Meanwhile, the LRP6 expression was evaluated, and the data confirmed that LRP6 was lowly expressed in the blood samples of patients with ischemic stroke (Figure 5e). To sum up, miR-29c-5p bound to LRP6 and negatively regulated the expression of LRP6 in astrocytes.

### 3.6 Rescue assay investigates the function of the miR-29c-5p/LRP6 axis in the TJP destruction of BMECs and inflammation caused by OGD-treated astrocytes

**Figure 6:**
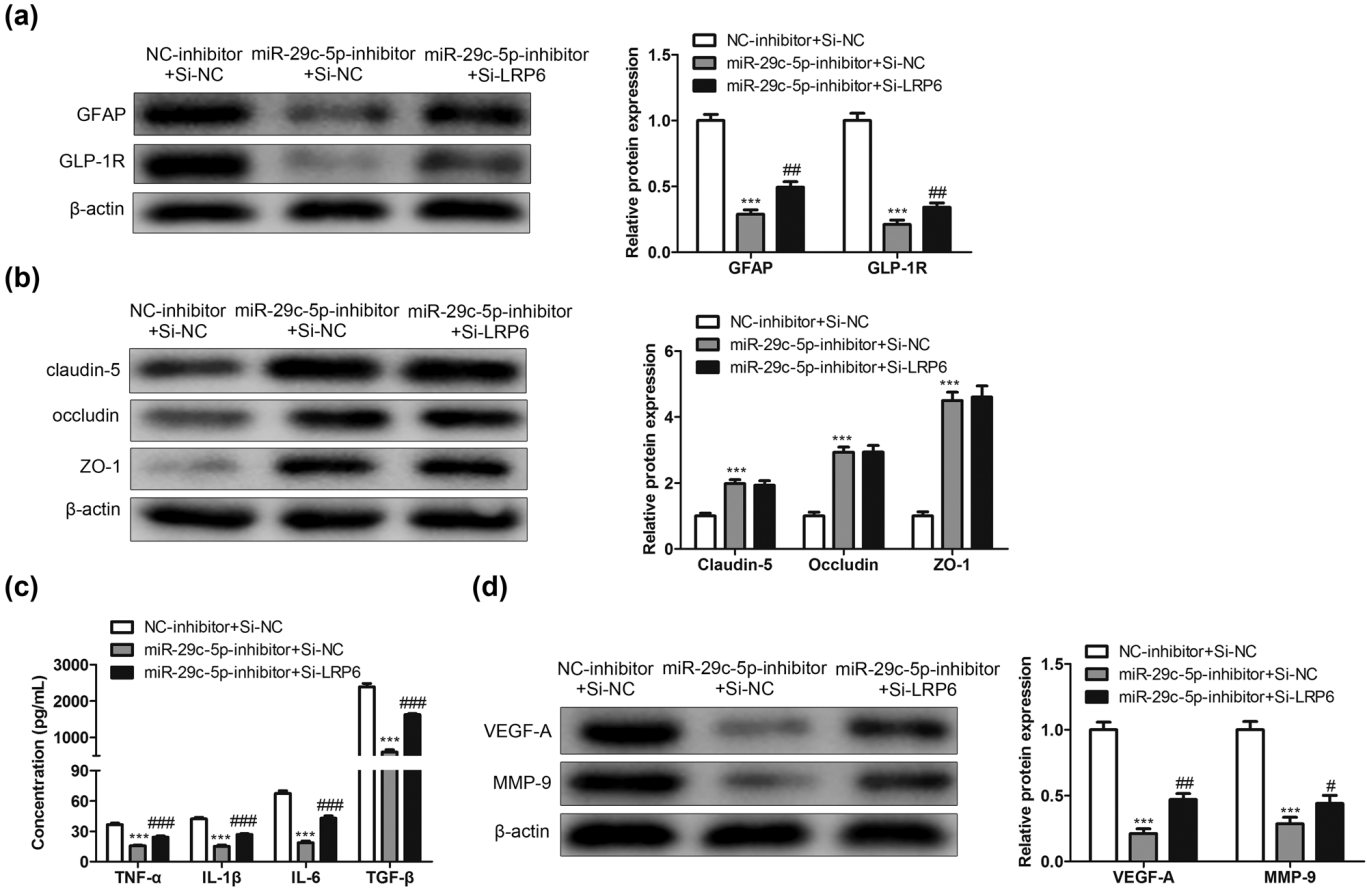
Verification of the regulatory function of the miR-29c-5p/LRP6 axis in the TJP destruction of BMECs and inflammation caused by OGD-treated astrocytes: (a) Astrocytes transfected with miR-29c-5p inhibitor and si-LRP6 were treated with OGD for 3 h. The concentrations of IL-1β, IL-6, TNF-α, and TGF-β in the cell culture supernatant of astrocytes were measured by ELISA. (b) Western blot was carried out to test the protein levels of VEGF-A and MMP-9. (c) Astrocytes transfected with miR-29c-5p inhibitor and si-LRP6 were treated with OGD for 3 h and then cocultured with BMECs for 24 h. The protein levels of GFAP and GLP-1R were quantified using Western blot. (d) Analysis of the claudin-5, occludin, and ZO-1 protein levels using Western blot. Data are presented as an average of three independent assays. ***P < 0.001 vs NC-inhibitor + Si-NC. ^#^ *P* < 0.05, ^##^ *P* < 0.01, ^###^ *P* < 0.001 vs miR-29c-5p inhibitor + si-NC.

We further investigated whether miR-29c-5p regulated the inflammation and TJP destruction of BMECs induced by OGD-treated astrocytes via LRP6. Astrocytes transfected with miR-29c-5p inhibitor and/or si-LRP6 were treated with OGD. As shown in Figure 6a, the interference with miR-29c-5p reduced the concentrations of IL-1β, IL-6, TNF-α, and TGF-β, and this reduction was compensated by the interference with LRP6. Similarly, the miR-29c-5p knockdown downregulated the protein levels of VEGF-A and MMP-9, whereas the LRP6 knockdown partially reversed this trend (Figure 6b). Furthermore, astrocytes transfected with miR-29c-5p inhibitor and si-LRP6 were treated with OGD and then cocultured with BMECs. From the results of Western blot, we found that the transfection of miR-29c-5p inhibitor downregulated the GFAP and GLP-1R protein levels, and this downregulation was partially reversed by the transfection of si-LRP6 (Figure 6c). Moreover, the interference with miR-29c-5p increased the protein levels of claudin-5, occludin, and ZO-1, whereas the interference with LRP6 partially reversed this increase (Figure 6d). Overall, the interference with miR-29c-5p ameliorated the inflammation and TJP destruction of BMECs induced by OGD-treated astrocytes by increasing LRP6.

## 4 Discussion

The previous study demonstrates that the miR-181 and miR-29 families are abnormally expressed in cerebral ischemia astrocytes [34]. Based on this, this study further verified the expressions of miR-181 family and miR-29 family in clinical blood samples from patients with ischemic stroke and authenticated that only miR-29c-5p was remarkably overexpressed. Thus, we focused on the mechanism of miR-29c-5p in ischemic stroke in the current study. Here, we demonstrated that the interference with miR-29c-5p decreased the high permeability and the TJP destruction of BMECs and the inflammation induced by OGD-treated astrocytes. Mechanism research results confirmed that miR-29c-5p interacted with LRP6 and negatively regulated LRP6 expression in astrocytes. Moreover, the rescue assays indicated that the interference with miR-29c-5p ameliorated the TJP destruction of BMECs and inflammation caused by OGD-treated astrocytes by upregulating LRP6 expression. Thus, our study might provide a novel regulatory axis for ischemic stroke: miR-29c-5p/LRP6.

Accumulated evidence suggest that BBB dysfunction is the pathogenesis of many neurological diseases, including ischemic stroke [35]. Various factors contribute to BBB dysfunction, including the major components of BBB: BMECs and astrocytes [6,36]. As reported, an *in vitro* BBB model composed of BMECs and primary culture of astrocytes has been used to elucidate the mechanisms of BBB and to develop novel therapeutic strategies [12]. Similarly, we obtained primary astrocytes from normal C57BL/6 mice and established a coculture system of BMECs and OGD-treated astrocytes. Our study confirmed that OGD treatment of astrocytes enhanced the permeability of BMECs. Based on this finding, we would continue investigating the potential mechanism of BMECs–astrocyte interaction under the pathological condition of BBB dysfunction after ischemic stroke.

Increasing studies illustrate that those different mechanisms, including miRNAs, are related to the pathogenesis of BBB dysfunction after ischemic stroke. For instance, Chen et al. identified a double-negative feedback loop: let-7i/TGF-βR1 in ischemic stroke, providing a potential therapeutic target for ischemic stroke [37]; Ma et al. indicated that the deletion of miR-15a/16-1 gene in endothelial cells represses BBB dysfunction after ischemic stroke, suggesting that miR-15a/16-1 mediates BBB dysfunction to alleviate ischemic stroke [38]. Similarly, our study illustrated that miR-29c-5p was elevated in clinical blood samples from patients with ischemic stroke, implying that miR-29c-5p might be related to the occurrence and development of ischemic stroke. Our functional study further clarified that the miR-29c-5p knockdown reduced the high permeability and the TJP destruction of BMECs and the inflammation induced by OGD-treated astrocytes.

As an essential member of the LRP family, LRP6 has been proved to exert an important regulatory function in the occurrence of ischemic stroke [21,39]. Interestingly, our research found that there were binding sites between miR-29c-5p and LRP6 through TargetScan and Metascape databases. Based on these findings, we further confirmed that miR-29c-5p bound to LRP6, and the expression of LRP6 in astrocytes was negatively regulated by miR-29c-5p, and LRP6 was lowly expressed in clinical blood samples from patients with ischemic stroke. Meanwhile, the rescue assays illustrated that the interference with miR-29c-5p ameliorated the TJP destruction of BMECs and inflammation caused by OGD-treated astrocytes by elevating LRP6.

In summary, our results provided evidence that miR-29c-5p improved the TJP destruction of BMECs and inflammation caused by OGD-treated astrocytes by upregulating LRP6. This study might provide a novel regulatory axis for ischemic stroke: miR-29c-5p/LRP6. Besides, there might also be other targets for miR-29C-5p, and we would continue to explore in-depth to further enrich the content of this study.
